# A new insight into role of phosphoketolase pathway in *Synechocystis* sp. PCC 6803

**DOI:** 10.1038/s41598-020-78475-z

**Published:** 2020-12-16

**Authors:** Anushree Bachhar, Jiri Jablonsky

**Affiliations:** Institute of Complex Systems, FFPW, University of South Bohemia, CENAKVA, Zamek 136, 373 33 Nove Hrady, Czech Republic

**Keywords:** Isoenzymes, Biochemical networks, Computational models

## Abstract

Phosphoketolase (PKET) pathway is predominant in cyanobacteria (around 98%) but current opinion is that it is virtually inactive under autotrophic ambient CO_2_ condition (AC-auto). This creates an evolutionary paradox due to the existence of PKET pathway in obligatory photoautotrophs. We aim to answer the paradox with the aid of bioinformatic analysis along with metabolic, transcriptomic, fluxomic and mutant data integrated into a multi-level kinetic model. We discussed the problems linked to neglected isozyme, *pket*2 (*sll0529*) and inconsistencies towards the explanation of residual flux via PKET pathway in the case of silenced *pket1* (*slr0453*) in *Synechocystis* sp. PCC 6803. Our in silico analysis showed: (1) 17% flux reduction via RuBisCO for Δ*pket1* under AC-auto, (2) 11.2–14.3% growth decrease for Δ*pket2* in turbulent AC-auto, and (3) flux via PKET pathway reaching up to 252% of the flux via phosphoglycerate mutase under AC-auto. All results imply that PKET pathway plays a crucial role under AC-auto by mitigating the decarboxylation occurring in OPP pathway and conversion of pyruvate to acetyl CoA linked to EMP glycolysis under the carbon scarce environment. Finally, our model predicted that PKETs have low affinity to S7P as a substrate.

## Introduction

Metabolic engineering of cyanobacteria provides many options for producing valuable compounds, e.g., acetone from *Synechococcus elongatus* PCC 7942^1^ and butanol from *Synechocystis* sp. strain PCC 6803^2^. However, certain metabolites or overproduction of intermediates can be lethal. There is also a possibility that required mutation(s) might be unstable or the target bacterium may even be able to maintain the flux distribution for optimal growth balance due to redundancies in the metabolic network, such as alternative pathways. The current dominant “trial and error” approach implies that our understanding of cellular metabolic regulatory mechanisms, including those involving isozymes, post-translational modifications or allosteric regulations, is secondary to achieve the desired effect/product. Integrating computational biology methods may help both to understand complex big data and to improve our understanding of cellular processes, improving potential biotechnological applications in the long term.

Cyanobacterium *Synechocystis* sp. strain PCC 6803 (hereafter referred to as *Synechocystis*) is one of the more complex prokaryotes, known for its metabolic plasticity^3^. All known glycolytic pathways that exist in nature are accommodated in its central carbon metabolism: the Embden–Meyerhof–Parnas (EMP) pathway, the oxidative pentose phosphate (OPP) pathway, the phosphoketolase (PKET) pathway and the Entner–Doudoroff (ED) pathway. However, our knowledge about regulatory roles of PKET in *Synechocystis* is still limited.

**Figure 1:**
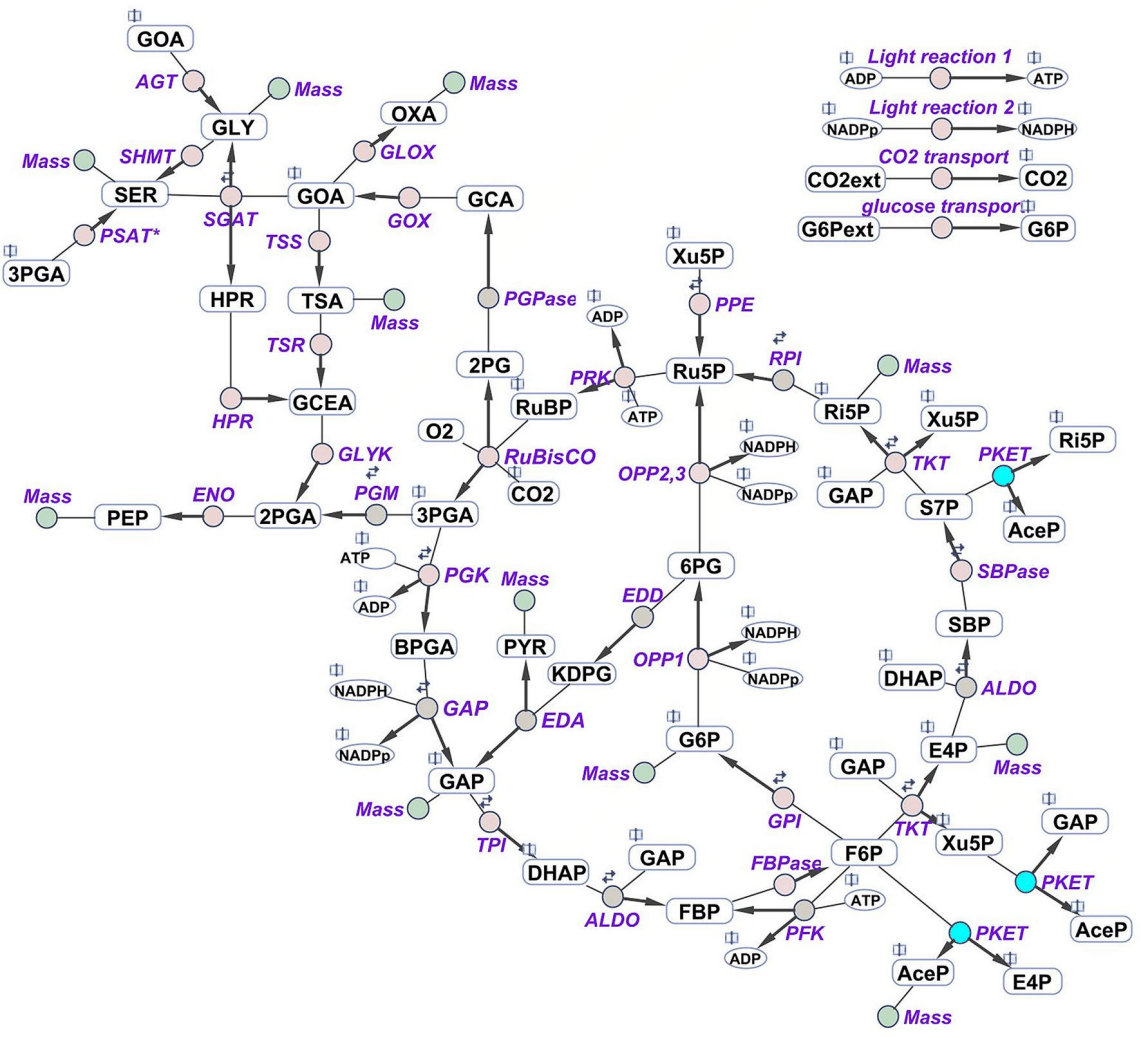
Schematic representation of the central carbon metabolism network, which was implemented in the multi-level kinetic model of *Synechocystis*. Blue indicates the reactions catalyzed by phosphoketolase. The model includes the Calvin-Benson cycle, glycogen synthesis (sink from glucose-6-phosphate), photorespiratory pathways, phosphoketolase pathway, glycolysis, the oxidative pentose pathway, Entner–Doudoroff pathway and sink reactions (representing the adjacent pathway and the calculation of biomass production). The reversibility of a particular reaction is indicated by two small arrows. Purple indicates the involved enzymes: *RuBisCO* ribulose-1,5-bisphosphate carboxylase oxygenase, *PGK* phosphoglycerate kinase, *GAP* glyceraldehyde-3-phosphate dehydrogenase, *TPI* triose-phosphate isomerase, *ALDO* aldolase, *FBPase* fructose-1,6 bisphosphatase, *PFK* phosphofructokinase, *TKT* transketolase, *SBPase* sedoheptulose-1,7 bisphosphatase, *RPI* phosphopentose isomerase, *PPE* phosphopentose epimerase, *PRK* phosphoribulokinase, *GPI* glucose-6-phosphate isomerase, *G6PD* glucose-6-phosphate dehydrogenase, *PGD* phosphogluconate dehydrogenase, *PGPase* phosphoglycolate phosphatase, *PKET* phosphoketolase, *GOX* glycolate oxidase, *SGAT* serineglyoxylate transaminase, *HPR* hydroxypyruvate reductase, *GLYK* glycerate kinase, *AGT* alanineglyoxylate transaminase, *TSS* tartronatesemialdehyde synthase, *TSR* tartronatesemialdehyde reductase, *SHMT* serine hydroxymethyltransferase, *GLOX* glyoxylate oxidase, PSAT* phosphoserine transaminase, *PGM* phosphoglycerate mutase, *ENO* enolase, *EDD* 6P-gluconate dehydratase, *EDA* 2-keto-3-deoxygluconate-6-phosphate aldolase (EDD and EDA are currently simplified into a single reaction in the model). Open book symbol indicates an involvement of metabolite in other reaction(s). The scheme was created in SimBiology toolbox of MATLAB 2010b (The MathWorks, Inc., Natick, Massachusetts, United States of America), http://www.mathworks.com.

Phosphoketolase converts xylulose 5-phosphate to glyceraldehyde 3-phosphate and acetyl phosphate, or, fructose 6-phosphate to erythrose 4-phosphate and acetyl phosphate, see Fig. 1. PKET is, based on current data (Uniprot, September 2020), present in around 98% of cyanobacteria, which makes it more common than phosphofructokinase (around 72%) or ED pathway (around 64%) (Uniprot, September 2020). PKET is found mostly in gram negative bacteria, cyanobacteria, fungi and algae but not in higher plants. It is assumed that PKET pathway plays a role in heterotrophic conditions^4^, and it is known that deactivation of PKET pathway leads to 11.5% slower growth under autotrophic ambient CO_2_ (AC-auto) condition^4^. Moreover, an engineered strain of *Synechocystis* with blocked glycogen synthesis and enabled xylose catabolism was reported with over 30% total carbon flux directed via PKET^4^ under mixotrophic condition on xylose and CO_2_. Furthermore, overproduction of acetyl phosphate, caused by overexpression of *pket* (PKET gene), can negatively influence the pentose sugar metabolism^5^ but may increase lipid synthesis^6^. Finally, recent ^13^C labelling study concluded that there is virtually zero flux via PKET under AC-auto^7^ in *Synechocystis*. This finding is in direct disagreement with the previous theoretical stoichiometric^8^ and kinetic^9^ studies which estimated the flux via PKET pathway to be 11.6% and 5.3% of flux via RuBisCO, respectively. Thus, we have decided to investigate this issue and to review the flux via PKET pathway based on all available data.

Our study aims to provide answers to the following questions with the help of multi-level kinetic model of central carbon metabolism for *Synechocystis*: (1) if PKET pathway is present in cyanobacteria, especially in obligatory photoautotrophs, why it is inactive in autotrophic conditions during day, (2) what is the role of PKET2 and can we quantify its potential benefits and (3) does the role of PKET pathway change in different environmental conditions?

## Results and discussion

### One or two phosphoketolases in *Synechocystis*?

**Figure 2:**
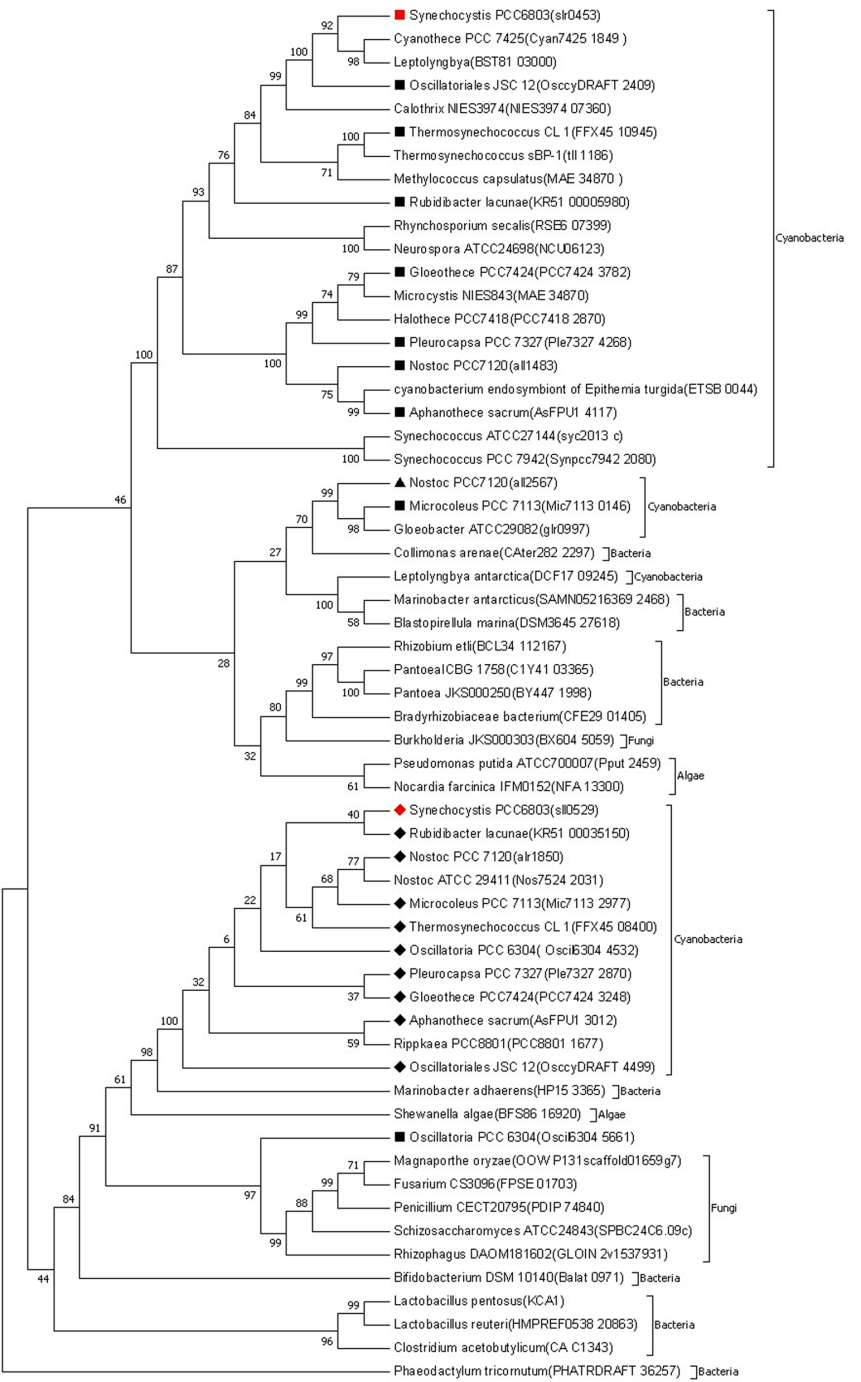
Phylogenetic tree created from the retrieved amino acid sequences of phosphoketolase (PKET) from sequence databases (Uniprot and NCBI) with MEGA 7 using Neighbour joining method (maximum bootstrap value of 500). The evolutionary distances were computed using the JTT matrix-based method. Square, diamond and triangle symbols are showing the first, second and third isozymes of PKET, respectively. Red colour is highlighting *Synechocystis*.

Existence of two PKETs was biochemically characterized in cyanobacteria so far only in *Nostoc/Anabena* PCC 7120 (Nostoc)^10^ but our multiple sequence alignment showed 78% homology among PKETs in cyanobacteria with annotated PKET isozymes (see Supplement). Phylogenetic analysis indicated a significant spread of PKET isozymes, see Fig. 2, namely a clearly separated cluster for cyanobacteria, containing also *pket1* (*slr0453*) of *Synechocystis* and pket1 (*all1483*) of Nostoc. Interestingly, the secondary PKETs of cyanobacteria, including *pket2* (*sll0529*) of *Synechocystis* and *pket2* (*alr1850*) of Nostoc, are clustered also with fungi, bacteria and algae, implying a possible horizontal gene transfer. The common feature of photosynthetic and non-photosynthetic species in this heterogenous cluster is a complex metabolism, allowing to survive on organic source of carbon. Strikingly, PKET2, firstly mentioned in 2010^11^, was ignored in the metabolic studies despite the residual (approximately 10%) flux via PKET pathway in the case of silenced *pket1* under both mixotrophic^4^ and autotrophic^7^ condition. It was suggested that this residual flux is caused by the activity of phosphate acetyl-transferase (PTA) activity^7^. The problem with this explanation is that it considers only reactions towards TCA cycle, i.e., reversibility of PTA, even though the same residual flux was detected also towards glyceraldehyde-3-phosphate (GAP) synthesis^4^. Since GAP synthesis is not connected to PTA activity (Fig. 1), the proposed role of PTA on the residual flux via PKET pathway lacks any justification.

We propose that neglected PKET2 of *Synechocystis* is responsible for the residual flux via PKET pathway. Furthermore, to explain why PKET2 is not taking over in the case of silenced *pket1*, we assumed that PKET2 might be allosterically inhibited. This prediction is supported by the fact that bacterial PKETs, clustered with PKET2 in question (Fig. 2), can be inhibited by several metabolites, including phosphoenolpyruvate, oxaloacetate and glyoxylate^12^. Moreover, based on residual flux, required 90/10 ratio of activity for PKET1 and PKET2 is not unique for *Synechocystis*. We know at least one more example of allosterically inhibited isozymes with similar activity ratio, the aldolase 1 and 2. Ninety percent of total aldolase activity involves aldolase 1 (class II) whereas aldolase 2 (class I) supplies only 10% of total due to allosteric inhibition^13^. It can thus be speculated that, after *pket1* is silenced, PKET2 is behind the residual activity of PKET pathway and PKET2 contribution does not increase due to allosteric inhibition. However, despite other known cases among isozymes in *Synechocystis*, allosteric inhibition is only one of possible explanations and we cannot prioritize it or exclude other options such as post-translational modification or regulation by signalling cascade(s), etc.

**Figure 3:**
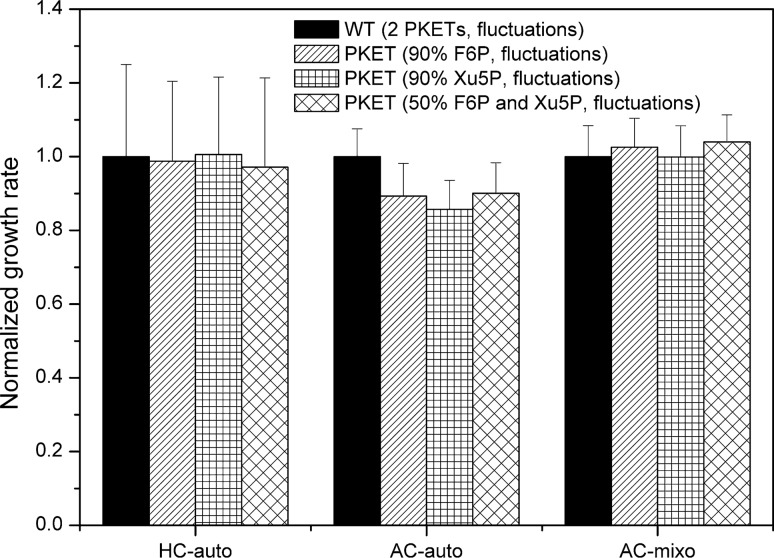
Comparing the impact of having one and two PKETs in the metabolism during simulated variation in gene expression in the different environments. AC-auto, HC-auto and AC-mixo denotes the growth conditions: ambient CO_2_ autotrophic, high CO_2_ autotrophic and ambient CO_2_ mixotrophic state, respectively. WT (wild type) represent the scenario with 2 PKETs. Single PKET scenarios indicate the same flux via PKET pathway as WT, however, with 90% preference of particular substrate (F6P or Xu5P) and 50% preference for both substrates. Fluctuation means a simulation of changing environment by random, up to ± 20%, variation in gene expression in the model. Each scenario was run 100-times; mean values and standard deviations are shown. We note that higher error bars for HC-auto indicates higher sensitivity towards changes in gene expression. Statistical analysis (paired t-test, 2 PKETs vs single PKET) showed no significance for HC-auto and AC-mixo (p > 0.05) but a great significance for AC-auto (p < 0.001).

We presented certain support for existence and role of PKET2. However, it is still unknown what, if any, are the benefits of secondary PKET in the metabolism or growth of *Synechocystis*. Fortunately, our method of multi-level kinetic modelling (see “Materials and methods”) is capable to quantify the impact of PKET2. We performed an in silico experiment in which growth behaviour of *Synechocystis* was simulated under turbulent environment mimicked by a random variation in gene expression, up to ± 20%. Two scenarios were tested, either one or two PKETs in the system; the flux via PKET pathway was same in both cases. Since it is unknown if either of F6P or Xu5P (Fig. 1) is preferred substrate for any specific growth condition, the fitted parameters for WT (i.e., 2 PKETs) could be biased. To avoid any mistake in judging the impact of single PKET in the system, we analysed two rather extreme cases (ninety percent of flux via PKET pathway occurs either via F6P branch or Xu5P branch) and balanced scenario (50% of flux occurs both via F6P branch and Xu5P branch). This analysis showed 11.2–14.3% higher growth in AC-auto in the case of 2 PKETs, see Fig. 3, regardless of low activity of PKET2. Furthermore, the negative impact of single PKET was highest for Xu5P as the preferred substrate and lowest for balanced distribution of substrates (Fig. 3). Interestingly, the impact of single PKET was positive but rather negligible under high CO_2_ autotrophic condition (HC-auto) and ambient CO_2_ mixotrophic condition (AC-mixo), varying slightly in dependence of preferred substrate (Fig. 3). However, the statistical analysis for HC-auto and AC-mixo indicated no significance for 2PKETs or a single PKET, i.e., second PKET can be viewed as expendable in these growth conditions.

In order to test the impact of PKET2 on growth in case of Δ*pket1*, the activity of original PKET pathway was reduced by 90%, based on the reported value of residual flux for Δ*pket1*. The simulated growth impact was − 13.6%, see Table 1, which was very close to previously reported experimental value − 11.5%^4^. We note that some changes in gene expression were probably induced in the Δ*pket1* mutant but could not be considered in the simulation due to the lack of available data. Furthermore, we showed that Δ*pket1* could be beneficial under HC-auto, see Table 1. Finally, the gene expressions of *pket1* and *pket2* were compared in the changing environment, see Table 2, and both were upregulated in AC-auto and downregulated in the in/organic carbon rich environment. All presented bioinformatical analyses and simulations lead to the following conclusions: (i) PKET2 is very likely to be present in *Synechocystis*, (ii) PKET2 has a positive impact on growth rate in turbulent, carbon scarce environment and (iii) PKET2 is currently the only explanation of the residual fluxes via PKET pathway in the case of silenced *pket1*, both towards TCA cycle and GAP synthesis.

**Table 1:** Impact of simulated silencing *pket1* on growth in various conditions.

	HC-auto	AC-auto	AC-mixo
Growth WT/Δ*pket1*	0.89	1.13	1.04

AC-auto, HC-auto and AC-mixo denotes the growth conditions, ambient CO_2_ autotrophic, high CO_2_ autotrophic and ambient CO_2_ mixotrophic state, respectively.

**Table 2:** Gene expression of phosphoketolases annotated for *Synechocystis*.

	AC-auto/HC-auto^1^	AC-auto/AC-mixo^2^
*pket1* (*slr0453*)	1.35	1.36
*pket2* (*sll0529*)	1.10	1.21

Transcriptomic data were taken from^16^ (1) and^18^ (2). AC-auto, HC-auto and AC-mixo denotes the growth conditions, ambient CO_2_ autotrophic, high CO_2_ autotrophic and ambient CO_2_ mixotrophic state, respectively.

### Flux contribution of phosphoketolase pathway in various scenarios

The first attempt to assess the flux via PKET pathway in *Synechocystis* was made with the aid of stoichiometric model based on flux balance analysis, suggesting flux of 14.9 (11.6% of flux via RuBisCO)^8^. At the time, it was believed that the model accurately describe HC-auto due to very good agreement with labeling experiment performed in HC-auto^14^. However, it was suggested later that the majority of fluxes are scalable in response to a changing environment^9^. Thus, explaining the stoichiometric model, designed for AC-auto, nearly fitting the fluxomic data for HC-auto. The rescaled value for PKET flux under AC-auto from stoichiometric model is then 4.1. This value is more than double of our previous estimate (1.9, 5.3% of flux via RuBisCO)^9^. However, ^13^C labeling experiment was recently performed for AC-auto which concluded virtually zero flux via PKET pathway^7^. This result implied that both theoretically estimated values of PKET flux were incorrect. The problems with such implication were: (1) stoichiometric modeling approach assumes the flux balance for maximal growth benefits so non-zero flux via PKET pathway indicates at least a potential growth benefit and (2) our previous kinetic model^9^, validated for different environmental conditions, also predicted non-zero flux via PKET pathway in order to improve the match between the experiments and simulations.

**Figure 4:**
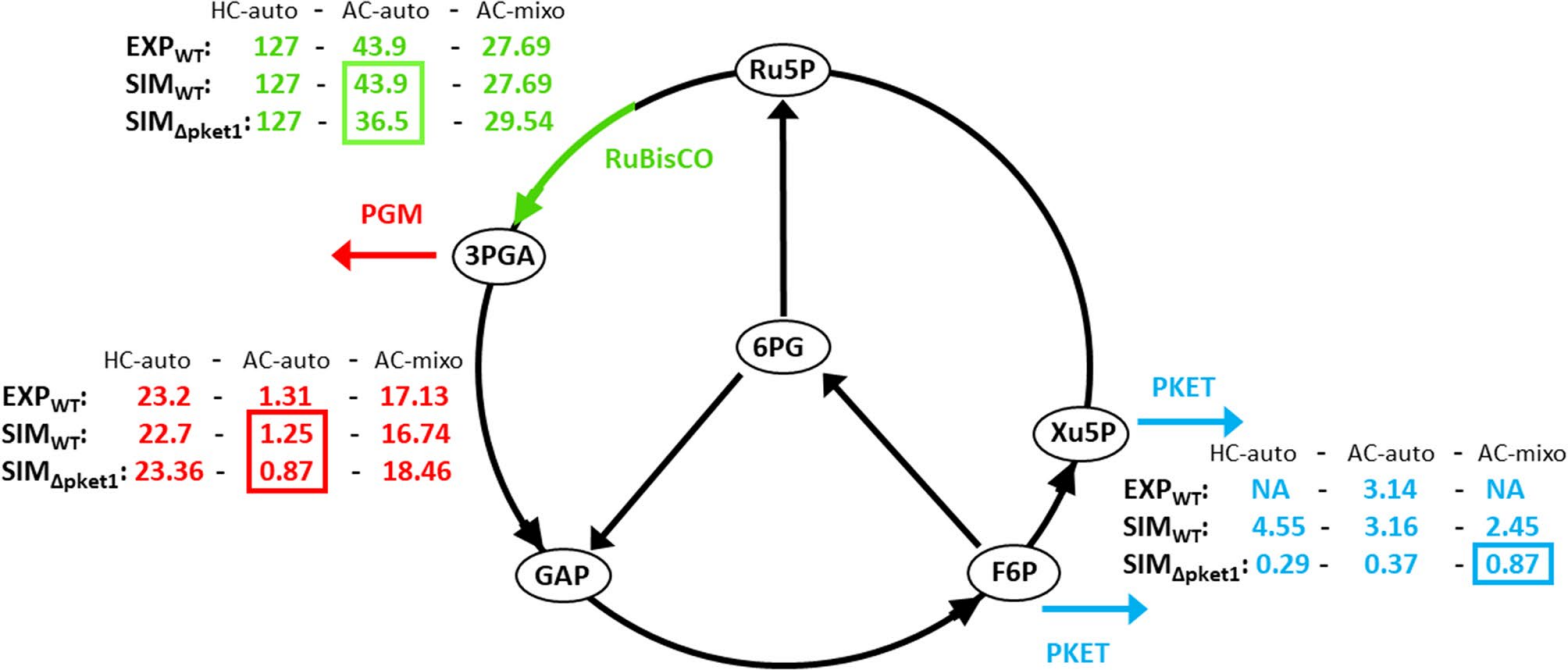
Experimental and estimated flux distribution in various environmental conditions. Green color indicates the flux via RuBisCO, blue color shows the flux via PKET (phosphoketolase) and red color highlights the flux via PGM (phosphoglycerate mutase). EXP_WT_ and SIM_WT_ indicates the experimental and simulated wild type values, respectively. SIM_ΔPKET1_ shows the results for silenced *pket1*, while PKET2 remains active. Marked values indicates interesting values or their significant changes. Sources of experimental fluxomic data: HC-auto^14^, AC-auto^7^ and AC-mixo^18^.

Therefore, we decided to elaborate the role and benefits of PKET pathway in detail. We tailored the flux via PKET1 and PKET2 to match 11.5% growth decrease under AC-auto for Δ*pket1*^3^. The simulated flux via PKET pathway was 3.16 (7.2% of flux via RuBisCO) under AC-auto which strongly out-performed (up to 2.5-fold) the flux via phosphoglycerate mutase, the exit point of EMP glycolysis from Calvin-Benson cycle, see Fig. 4; we note the high upper bound for PGM in the original labeling study^7^. Since more accurate simulation actually increased the estimated flux via PKET pathway, we compared these values with original fluxes from labeling experiment^7^. The data from labeling experiment calculated a virtually non-existent flux via Xu5P branch (10^–6^) and high flux (16.86, 7.2% of flux via RuBisCO) via F6P branch of PKET pathway^7^ which agrees with our predicted value perfectly. However, this result was not mentioned in their discussion, probably due to zero lower bound value for flux via both branches of PKET pathway, implying theoretically inactive PKET pathway. The lower bound of PKET flux might be explained only by reversibility of PTA. In the case of PTA flux, lower bound was 16.86 which was the same as calculated flux via F6P branch of PKET pathway. Since we have already rejected the reversibility of PTA as the source of residual flux via PKET pathway (see the section above), we can conclude that the flux via PTA is mostly, if not fully, originated from PKET pathway.

Our flux analysis revealed other interesting information. First of all, silencing *pket1* has negative impact over all fluxes, especially via RuBisCO, under AC-auto, see Fig. 4. Hence, despite the carbon flux redirection after silencing *pket1*, less amount of carbon is available in the system due to decarboxylation in OPP and conversion of pyruvate to acetyl CoA linked to EMP glycolysis. This conclusion is further supported by the data from AC-mixo (Fig. 4) where we can see that silencing *pket1* has a positive effect on fluxes via RuBisCO and PGM, i.e., the flux redirection is beneficial and is not impaired be limited carbon resources. Finally, we can see that impact of silenced *pket1* on the residual flux is different under all environmental conditions; namely that the residual flux in AC-mixo is 35.5% of WT flux which could be due to significant change in metabolic network fluxes, substrates and inhibitors concentration in the presence of glucose, some of which are not considered in the model.

### Sedoheptulose-7P—an overlooked substrate of PKET?

Recently, sedoheptulose-7P (S7P) has been identified as a substrate of PKET in *E. coli* although with very low affinity (K_M_ = 68.1 mM) and only 0.42% of total PKET activity maintained by S7P branch^15^. PKET breaks down S7P into ribose-5P and acetyl-P (Fig. 1). However, no information regarding S7P branch of PKET is available for cyanobacteria where it might play a more prominent role. Therefore, we designed an in silico experiment to predict the possible benefits of this pathway on growth by maximizing the expression of *pket1* and *pket2*. Our growth-based in silico analysis predicted that PKET pathway with S7P as one of substrates is once again beneficial only for AC-auto condition (data not shown). The target of our in silico experiment was to maximize the growth with any combination of the substrates (F6P, Xu5P and S7P) for PKETs. Firstly, the statistical analysis of results implies significantly higher variability in growth rate when S7P is metabolized via PKET pathway (Table 3). When the best fits were compared, 4.5% decline in growth was observed when S7P was metabolized by PKETs (Table 3). Thus, involvement of S7P in PKET pathway is not providing any robustness to the system but rather has a destabilizing effect on the metabolic homeostasis; one of the possible explanations could be competition for S7P with transketolase. Although the estimated growth based on experimental data (Δ*pket1*) is the same for both scenarios (with or without S7P), the reason behind the same value is that parameter estimation routine applied zero flux via S7P branch of PKET pathway due to its lower efficiency. Thus, our model predicts that the affinity of PKETs towards S7P in cyanobacteria is very low, similarly as shown for *E. coli*^15^. Nevertheless, experimental verification is required as the role of S7P branch of PKET pathway might be more significant under certain stress conditions.

**Table 3:** In silico analysis of substrates dependent tuned PKET pathway for theoretical maximal growth under AC-auto in *Synechocystis*.

Substrates of PKET	Growth in AC-auto			
	Mean	SD	Highest value	Data fit
F6P and Xu5P	118.52	1.08	119.07	110.73
F6P, Xu5P and S7P	100	13.48	113.69	110.73*

Mean and SD represent the statistical data from parameter estimation. Highest growth value denotes the maximal theoretical growth for a particular set of substrates. Data fit indicates the estimated growth rate based on experimental data (Δ*pket1*). Asterisks signifies data fit for scenario with S7P allowed to be metabolized by PKETs but the best fit considered zero activity via S7P branch.

## Conclusions

We presented the following support for the existence and role of two PKETs in *Synechocystis*: (1) bacteria have PKETs that are regulated by allosteric inhibition^12^ and *pket2* is in the cluster with bacteria (in contrast to *pket1*), opening the possibility for horizontal gene transfer; thus we can speculate that PKET2 is also allosterically inhibited by phosphoenolpyruvate, oxaloacetate or glyoxylate which provides currently the only explanation for the residual flux via PKET pathway in Δ*pket1* and clarifies why PKET2 does not display any higher activity in such scenario; however, other alternative explanations such as post-translational modification cannot be currently excluded, (2) our in silico experiment of Δ*pket1* mutant with active PKET2 showed the same impact on growth as reported previously in AC-auto for silenced *pket1* which was believed at the time to be a total inhibition of PKET pathway, (3) there are other known cases of allosteric inhibition in *Synechocystis*, including the proposed 90/10 flux contribution of isozymes, e.g., aldolase 1 and 2, and (4) we showed a clear benefit of having PKET2 in the central carbon metabolism of *Synechocystis*, namely, an 11.2–14.3% growth increase in the turbulent AC-auto. However, an experimental verification, if the product of gene *sll0529* (*pket2*) indeed has phosphoketolase activity, as well as what, if any, is the impact of phosphate acetyl-transferase on residual flux via PKET pathway, is needed. Similarly, here proposed allosteric regulation of PKET should be experimentally verified.

In addition to analysing the putative PKET2 isozyme, we have also focused on the flux distribution of PKET pathway under various conditions. We showed that PKET pathway could be the major carbon flux exit point from Calvin–Benson cycle in AC-auto, overcoming the flux via phosphoglycerate mutase by up to 252%. This finding is in direct disagreement with recent ^13^C labelling experiment which revealed virtually no flux via PKET pathway under AC-auto. However, we have found a critical error in their analysis and their own labelling data supports our conclusion of the great importance of PKET pathway. Furthermore, we have shown that silencing *pket1* has a negative impact over all fluxes, including RuBisCO and PGM in AC-auto but not in HC-auto and AC-mixo. We conclude that high flux via PKET pathway mitigates the decarboxylation occurring in OPP pathway and conversion of pyruvate to acetyl CoA linked to EMP glycolysis under AC-auto, i.e., in the carbon scarce environment. Finally, we have tested S7P as a substrate for PKETs as it was reported for *E. coli*. Our analysis predicted low efficiency of S7P branch of PKET pathway and low affinity towards S7P, similarly as shown for *E. coli*.

In the end, we did not analyse the role of PKET pathway under heterotrophic condition because its impact has already been shown and because heterotrophic condition is substantially more difficult to integrate into our light-on-only multi-state model. Our main goal was to address the purported negligible impact of PKET pathway in autotrophic condition; however, we plan to analyse the role of PKET in heterotrophic or photoheterotrophic conditions in the future, together with other isozymes within the central carbon metabolism of *Synechocystis*.

## Materials and methods

### Sources of experimental data integrated into multi-level kinetic model

Transcriptomic data

- HC-auto to AC-auto^16^.
- AC-auto to AC-mixo^17^.

^13^C labeling data

- HC-auto^14^.
- AC-auto^7^.
- AC-mixo^18^.

We note that, despite its not clearly stated in the original work as authors are talking only about photoautotrophic condition^14^, there is a line of evidence that this particular labeling experiment is describing HC-auto. Firstly, the growth rate of culture was 0.09 h^−1^^14^ which is higher rate than the one observed for 3% CO_2_ enriched medium (0.06 h^−1^)^7^. Secondly, authors used a high concentration of bicarbonate in their study and demonstrated that level of photorespiration is very small under high CO_2_ conditions^14^; study was performed under single environmental condition, i.e., HC-auto. Finally, we showed that majority of metabolic fluxes scales based on the level of carbon^9^ which explains why most of fluxes are very similar for AC-auto and HC-auto after normalization. However, key exit fluxes, such as phosphoglycerate mutase, OPP or PKET pathway do not scale^9^ which explains significant difference for exit fluxes between^14^ and^7^, e.g., ratio between fluxes via RuBisCO and OPP is 7.8 for HC-auto^14^ but 1.7 for AC-auto^7^.

Metabolic data

- HC-auto^19–21^.
- AC-auto^19,20^.
- AC-mixo^17^.

Mutant data

- Δ*pket1* mutant^4^.

### General information about the model

The multi-level kinetic model for *Synechocystis* was developed and simulations were executed using the SimBiology toolbox, Optimization toolbox, Global optimization toolbox and Parallel toolbox of MATLAB (The MathWorks, Inc., Natick, Massachusetts, United States of America). The routine for parameter estimation was a hybrid genetic algorithm. The model for HC-auto is available in the Supplement in SBML format L2V4 compatible with MATLAB 2010b and higher. We recommend to open the model either in MATLAB or user friendly COPASI (free academic license). The weight factors (applied transcriptomic data) necessary to run the model in other conditions (AC-auto and AC-mixo) is provided in Supplement.

The scope of the model includes the following parts of central carbon metabolism: Calvin–Benson cycle, photorespiration, all glycolytic pathways (Embden–Meyerhof–Parnas pathway, Entner–Doudoroff pathway, phosphoketolase pathway and oxidative pentose phosphate) and carbohydrate synthesis. These metabolic reactions were coupled to simplified light reactions and Ci and glucose uptake as the main input parameters. Biomass production is estimated as weighted sum of sink reactions^9^. This simplified approach allowed to estimate the CO_2_ fixation and glucose uptake by comparing to relative experimental fluxes in all tested conditions as well as to keep the track of growth rate, matching the experimental levels from literature^9^. Moreover, the biomass in the model is assumed to be equal to the amount of fixed carbon, whereas nitrogen was not a limiting nutrient factor in the experimental setups^9^. The definition of biomass in the model is provided by following equation, based on the model content, in which upper index *s* stands for accumulation of respective metabolite in the sink (Fig. 1) and a particular coefficient corresponds to the amount of carbon atoms in the molecule:

$$ {\text{Mass}} = 2*({\text{AceP}}^{{\text{s}}} + {\text{GLY}}^{{\text{s}}} + {\text{OXA}}^{{\text{s}}} ) + 3*({\text{PEP}}^{{\text{s}}} + {\text{PYR}}^{{\text{s}}} + {\text{GAP}}^{{\text{s}}} + {\text{SER}}^{{\text{s}}} ) + 4*{\text{E}}4{\text{P}}^{{\text{s}}} + 5*{\text{Ri}}5{\text{P}}^{{\text{s}}} + 6*{\text{G}}6{\text{P}}^{{\text{s}}} . $$

The model consists of 61 reactions, 48 metabolites and 214 kinetic parameters. The enzymatic reactions are described by Michaelis–Menten kinetics, except for the light reactions, Ci and glucose uptake, which are described by mass action kinetics. All reactions, except for the import of external carbon, are localized in a single compartment. The starting point of modelling was our previous model of *Synechocystis*^9^, which was modified and extended in order to describe AC-mixo condition. We note that the justification of using fold changes in the mRNA levels as a proxy between different environmental conditions, assuming a 1:1 ratio between a change in transcriptome and enzymatic amount, is provided in our previous study, including the support from experimental studies, can be found in our previous study^9^.

### Systems biology workflow

The integration of various omics data occurs in several steps. In the first step, transcriptomic data from HC-auto to AC-auto and AC-auto to AC-mixo are included into the model in the form of mean values of measured mRNA changes as weight factors for each V_max_. Then, we fit the experimental fluxomics and metabolic data from HC-auto. In this process of parameter estimation, we obtain a set of parameters which will be used in all following steps or trashed and search for new set will begin. If there is a match between simulation and experiment in HC-auto, metabolic and fluxomics data are saved (including the set of kinetic parameters). Then, we simulate a transition from HC-auto to AC-auto, i.e., applying the weight factors (mRNA), run the simulation for the steady state AC-auto, save the metabolic and fluxomic data and do the same for AC-auto to AC-mixo shift. At this point, we do semi-automated check for the reasonable match between simulated and experimental data for AC-auto and AC-mixo (growth levels, physiological range of key metabolites such as 3PGA, etc.), followed by manual curation of results. If acceptable match is found, the final step is a fine tuning of parameters and observing the changes in all three conditions. Manual tuning and curation of kinetic parameters at the different levels of parameter estimation and data integration is an essential part of modelling, because it provides a deeper understanding of system behavior and helps to detect errors in the algorithms, routines and model structure, e.g., when a new pathway is added.

We note that we shifted our priority from metabolic levels to fluxomic data as a key reference for match fitting. The main reason is that we are analyzing now 3 environmental conditions and planning to add more. However, there is no study providing metabolic data for the particular conditions of our interest and combining multiple metabolic data sets from different studies proved to be extremely challenging. We continue observing and analyzing the metabolic levels but in the case of parameter fitting, we currently only check if metabolic levels are in the physiological range and analyze the homeostatic stability of metabolites. Finally, simulation of fluctuating environment was done by applying up to ± 20% random variation in all V_max_ and k_f_ parameters, running it 100-times and calculating the mean value and standard deviation. Two scenarios, with one or two PKET, were tested, in all three environmental conditions.

More information about the method, parameter estimation and other details can be found in our previous study^9^. The first application of the method, the case study of phosphoglycerate mutase, discussed the potential of this method, e.g., the verification of gene annotation^22^.

## Supplementary Information

Supplementary Information 1.

Supplementary Information 2.

Supplementary Information 3.

Supplementary Information 4.

Supplementary Information 5.
