# Supplementary material for: A new insight into role of phosphoketolase pathway in Synechocystis sp. PCC 6803
Source: Sci Rep. 2020 Dec 16;10:22018. doi: 10.1038/s41598-020-78475-z (PMC7744508; doi:10.1038/s41598-020-78475-z)

Supplementary Info File

A new insight into role of phosphoketolase pathway in Synechocystis sp. PCC 6803

Anushree Bachhar, Jiri Jablonsky

Multiple sequence alignment for phosphoketolase in cyanobacteria

Legend:

The multiple sequence alignment of phosphoketolase isoenzymes curated from UniProt database (https://www.uniprot.org/uniprot/?query=taxonomy:1117%20phosphoketolase%20taxonomy:cyanobacteria). The sequence alignment was performed in multiple sequence alignment tool clustal omega (https://www.ebi.ac.uk/Tools/msa/clustalo/) using clustal W platform. The output was edited using Jalview software to identify the conserved region (in blue) based on BLOSUM62 matrix based score. The sequences for Synechocystis PKET1 (slr0453) and PKET 2 (sll0529) isoenzymes were highlighted in red.

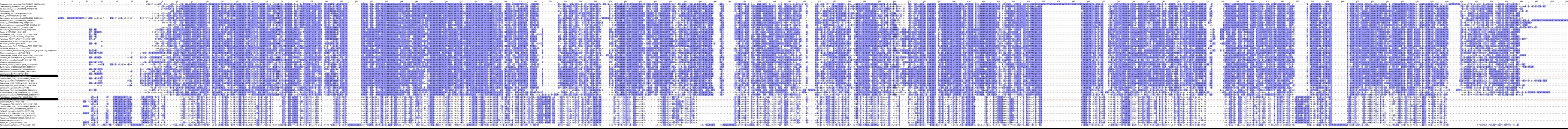

Supplement: Supplementary file 2 — Supplementary Information 2. [file 41598_2020_78475_MOESM2_ESM.pdf]
