# Supplementary material for: A new insight into role of phosphoketolase pathway in Synechocystis sp. PCC 6803
Source: Sci Rep. 2020 Dec 16;10:22018. doi: 10.1038/s41598-020-78475-z (PMC7744508; doi:10.1038/s41598-020-78475-z)
Supplement: Supplementary file 5 — Supplementary Information 5. [file 41598_2020_78475_MOESM5_ESM.docx]

**Supplementary data**

1. Multiple sequence alignment for phosphoketolase in cyanobacteria
2. Sequence information used for creation of presented phylogenetic tree.
3. mRNA fold changes from various environmental conditions employed as weight factors for Vmax parameters
4. Kinetic model of central carbon metabolism for *Synechocystis* in .xml format
